# The Inhibition of Polo Kinase by Matrimony Maintains G2 Arrest in the Meiotic Cell Cycle

**DOI:** 10.1371/journal.pbio.0050323

**Published:** 2007-12-04

**Authors:** Youbin Xiang, Satomi Takeo, Laurence Florens, Stacie E Hughes, Li-Jun Huo, William D Gilliland, Selene K Swanson, Kathy Teeter, Joel W Schwartz, Michael P Washburn, Sue L Jaspersen, R. Scott Hawley

**Affiliations:** 1 Stowers Institute for Medical Research, Kansas City, Missouri, United States of America; 2 University of Kansas School of Medicine, Kansas City, Kansas, United States of America; Whitehead Institute, United States of America

## Abstract

Many meiotic systems in female animals include a lengthy arrest in G2 that separates the end of pachytene from nuclear envelope breakdown (NEB). However, the mechanisms by which a meiotic cell can arrest for long periods of time (decades in human females) have remained a mystery. The *Drosophila* Matrimony (Mtrm) protein is expressed from the end of pachytene until the completion of meiosis I. Loss-of-function *mtrm* mutants result in precocious NEB. Coimmunoprecipitation experiments reveal that Mtrm physically interacts with Polo kinase (Polo) in vivo, and multidimensional protein identification technology mass spectrometry analysis reveals that Mtrm binds to Polo with an approximate stoichiometry of 1:1. Mutation of a Polo-Box Domain (PBD) binding site in Mtrm ablates the function of Mtrm and the physical interaction of Mtrm with Polo. The meiotic defects observed in *mtrm/+* heterozygotes are fully suppressed by reducing the dose of *polo^+^*, demonstrating that Mtrm acts as an inhibitor of Polo. Mtrm acts as a negative regulator of Polo during the later stages of G2 arrest. Indeed, both the repression of Polo expression until stage 11 and the inactivation of newly synthesized Polo by Mtrm until stage 13 play critical roles in maintaining and properly terminating G2 arrest. Our data suggest a model in which the eventual activation of Cdc25 by an excess of Polo at stage 13 triggers NEB and entry into prometaphase.

## Author Summary

Many meiotic systems in females animals include a lengthy arrest period (spanning days in flies and to decades in humans) that separates the early and late stages of meiosis. Such an arrest raises the question: how can the quiescent meiotic cell cycle be precisely awakened or re-started? At least in principle, the answer to this phenomenon, which we refer to as “The Sleeping Beauty Kiss,” might have two molecular solutions: the controlled expression of a protein that re-starts the cell cycle, or the inactivation of an inhibitory protein that prevents such a re-start. We show here that the re-start of the meiotic cycle in *Drosophila* depends on both mechanisms: the controlled expression of an “activator” known as Polo kinase, and the presence of a regulatory protein called Matrimony (Mtrm), which binds to and physically inactivates Polo. Indeed, Mtrm is the first known protein inhibitor of Polo kinase. The excess of Mtrm prior to the time of normal meiotic re-start, keeps Polo inactive. However, either the production of an excess quantity of Polo, or the destruction of Mtrm, at the appropriate time, releases active Polo, permitting a properly controlled re-start of meiotic progression.

## Abstract 3

The restart of the meiotic cycle in *Drosophila* depends on both the controlled expression of Polo kinase and a protein called Matrimony (Mtrm), which binds to and physically inactivates Polo.

## Introduction

**Figure 1:**
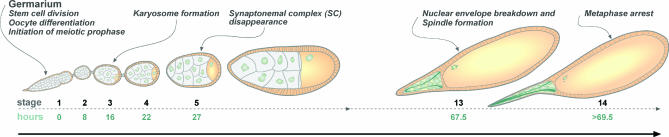
Oocyte Development in *D. melanogaster* This figure displays a schematic depiction of oocyte development showing the timing (in hours) of the relevant stages. The end of meiotic prophase, as defined by SC dissolution, occurs at stages 5–6. By the end of stages 5–6, the chromosomes have condensed into a dense mass known as the karyosome, as pointed out by Mahowald and Kambysellis [2]. The karyosome remains compacted until stages 8–10, at which time it decondenses and a high level of transcription is observed. The chromosomes recompact during stages 11 and 12 to form a tight mass that is released into the cytoplasm upon NEB at stage 13. The end of pachytene is separated from NEB by approximately 40 h.

The mechanism of the lengthy arrest in G2 that separates the end of pachytene from nuclear envelope breakdown (NEB)—which is a characterization of many female meiotic systems—has remained a mystery. One can imagine that both the maintenance and the termination of this arrest might involve either or both of two mechanisms— the transcriptional or translational repression of a protein that induces NEB, and thus meiotic entry, or the presence of an inhibitory protein that precludes entry into the first meiotic division. Because *Drosophila* females exhibit a prolonged G2 arrest (see Figure 1) and are amenable to both genetic and cytological analyses, they provide an ideal system in which to study this problem.

The ovaries of *Drosophila* females are composed of a bundle of ovarioles, each of which contains a number of oocytes arranged in order of their developmental stages [1–3]. For our purposes, the process of oogenesis may be said to consist of three separate sets of divisions: the initial stem cell divisions, which create primary cystoblasts; four incomplete cystoblast divisions, which create a 16-cell cyst that contains the oocyte; and the two meiotic divisions. Although a great deal is known regarding the mechanisms that control cystoblast divisions and oocyte differentiation, relatively little is known about the mechanisms by which the progression of meiosis is controlled.

As is the case in many meiotic systems, female meiosis in *Drosophila* involves preprogrammed developmental pauses. The two most prominent pauses during *Drosophila* meiosis are an arrest that separates the end of pachytene at stages 5–6 from NEB at stage 13, and a second pause that begins with metaphase I arrest at stage 14 and continues until the egg passes through the oviduct. It is the release of this second preprogrammed arrest event that initiates anaphase I and allows the completion of meiosis I followed by meiosis II. As shown in Figure 1, the end of meiotic prophase by dissolution of the synaptonemal complex (SC) at stages 5–6 [4,5] is separated from the beginning of the meiotic divisions, which is defined by NEB at stage 13, by approximately 40 h to allow for oocyte growth.

We are interested in elucidating the mechanisms that arrest meiotic progression at the end of prophase, but then allow onset of NEB and the initiation of meiotic spindle formation some 40 h later. One intriguing possibility is that during this period of meiotic arrest, the oocyte actively blocks the function of cell cycle regulatory proteins such as cyclin dependent kinase 1 (Cdk1), the phosphatase Cdc25, and Polo kinase (Polo), all of which promote meiotic progression just as they do during mitotic growth. Recently, Polo was shown to be expressed in the germarium and required for the proper entry of *Drosophila* oocytes into meiotic prophase, as defined by the assembly of the SC [6]. Decreased levels of Polo resulted in delayed entry into meiotic prophase, whereas overexpression of Polo caused a dramatic increase in the number of cystocyte cells entering meiotic prophase, indicating that Polo is involved both in the initiation of SC formation and in the restriction of meiosis to the oocyte. How then is Polo, which is known to play multiple roles in promoting meiotic and mitotic progression [7,8], prevented from compelling the differentiated oocyte to proceed further into meiosis?

One component of this regulation may well lie in the fact that Polo is not expressed during much of oogenesis. As shown below, Polo is clearly visible in the germarium but is then absent until stage 11, when it begins to accumulate to high levels in the oocyte (Figure S1). We show here that a second component of Polo regulation is mediated by binding to the protein product of the *matrimony* (*mtrm*) gene, which occurs from stage 11 until the onset of NEB at stage 13. This binding serves to inhibit Polo in the early stages of its expression, and thus prevents precocious nuclear envelope breakdown.

The *mtrm* gene was first identified in a deficiency screen for loci that were required in two doses for faithful meiotic chromosome segregation [9]. *mtrm/+* heterozygotes display a significant defect in achiasmate segregation (the meiotic process that ensures the segregation of those homologs that, for various reasons, fail to undergo crossingover). As a result of this defect, *mtrm/+* heterozygotes exhibit high levels of achiasmate nondisjunction. As homozygotes, *mtrm* mutants are fully viable but exhibit complete female sterility. We show here that the Mtrm protein prevents precocious NEB. Indeed, as discussed below, the effects of reducing the dose of *mtrm* on meiotic progression and on chromosome segregation are easily explained as the consequence of precocious NEB at stages 11 or 12, and can be suppressed by simultaneously reducing the copy number of *polo^+^*. In addition, the effects of heterozygosity for loss-of-function alleles of *mtrm* can be phenocopied by increasing the copy number of *polo^+^*. These genetic interactions suggest that Mtrm negatively regulates Polo in vivo.

Interestingly, Mtrm was shown to interact physically with Polo by a global yeast two-hybrid study [10]. We demonstrate that this yeast two-hybrid finding reflects a true physical interaction in vivo by both coimmunoprecipitation studies and by multidimensional protein identification technology (MudPIT) mass spectrometry experiments, which indicate that Mtrm binds to Polo with an approximate stoichiometry of 1:1. Moreover, ablating one of the two putative Polo binding sites on Mtrm by mutation prevents the physical interaction between Polo and Mtrm and renders the mutated Mtrm protein functionless. This experiment, along with genetic interaction studies, provides compelling evidence that the function of the binding of Mtrm to Polo is to inhibit Polo, and not vice versa.

The analysis of *mtrm* mutants allows us to examine the effects of premature Polo function during oogenesis. Our evidence shows that in the absence of Mtrm, newly synthesized Polo is capable of inducing NEB from stage 11 onward. As a result of this precocious NEB, chromosomes are not properly compacted into a mature karyosome and they are released prematurely onto the meiotic spindle. In many cases, the centromeres of achiasmate bivalents subsequently fail to co-orient.

## Results

### The *mtrm* Gene Encodes a 217–Amino Acid Protein Whose Expression Is Limited to the Period between the End of Pachytene and the Onset of NEB

**Figure 2:**
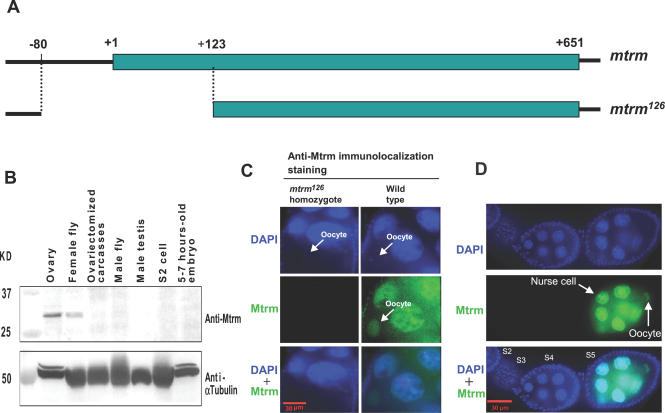
The *mtrm* Gene and Its Expression Pattern (A) Schematic diagram of the 651-bp *mtrm* gene. The *mtrm^126^* deletion allele, which was created by imprecise excision of the P element insertion mutation *KG08051*, is deleted for 203 bases (80 bases upstream of the first ATG in *mtrm* and 123 downstream of that ATG). (B) Mtrm is expressed exclusively in ovaries. Protein extracts from the indicated tissues were analyzed by Western blotting using an antibody to Mtrm. These experiments reveal that Mtrm, a 27-kDa protein, is expressed only in ovaries. The lower panel displays a Western blot of equal amounts of protein from the same extracts probed with antibody to alpha-tubulin (50-kDa). (C) Immunostaining using the antibody to Mtrm to stage 9 oocytes reveals that Mtrm is expressed in the nuclei of both oocytes and nurse cells in wild-type egg chambers but not in *mtrm* homozygote egg chambers. The latter finding indicates the antibody to Mtrm is indeed specific to Mtrm. (D) Timing of Mtrm expression during oocyte development. Endogenous Mtrm expression is not detectable before stage 5. At stage 5, Mtrm localizes to both the oocyte and nurse cells. Scale, 30 μm.

The *mtrm* gene was first identified as a dosage-sensitive meiotic locus; heterozygosity for a loss-of-function allele of *mtrm* specifically induced the failed segregation of achiasmate homologs [9]. The *mtrm* gene encodes a 217–amino acid protein with two Polo-Box Domain (PBD) binding sites (STP and SSP) and a C-terminal SAM/Pointed domain. The studies reported in this paper rely primarily on a null allele of *mtrm* (*mtrm^126^*), which removes 80 bp of upstream sequence and the sequences encoding the first 41 amino acids of the Mtrm protein (Figure 2A).

Western blot analysis using an antibody to Mtrm reveals that Mtrm can only be detected in ovaries (Figure 2B). This is consistent with a previous report by Arbeitman et al**.** [11], which showed that the expression profile of the *mtrm* gene product was strictly maternal and that its expression was reduced greater than 10-fold over 0–6.5 h of embryonic development. The specificity of this antibody is demonstrated by the fact that no signal was detected by either Western blotting or by immunofluorescence of ovarioles homozygous for the *mtrm^126^* mutant (Figure 2C). Immunofluorescence studies using the same antibody reveal that Mtrm is expressed as a diffuse nuclear protein in the oocytes and nurse cells beginning at stage 4–5 (Figure 2C and 2D*).* As shown in Figure 2C, the Mtrm signal was not restricted to the karyosome itself; but rather Mtrm seems to fill the space in the entire nucleus. Although Mtrm is restricted to the nucleus until approximately stage 10, it localizes throughout the oocyte in later stages. Mtrm brightly stains both the oocyte nucleus and cytoplasm between stage 11 and stage 12, but staining is greatly reduced at stage 13, the stage at which NEB occurs (Figure S1).

### Reducing the Dose of the *polo^+^* Gene Suppresses the Chromosome Segregation Defects Observed in *mtrm/+* Heterozygotes

**Figure 3:**
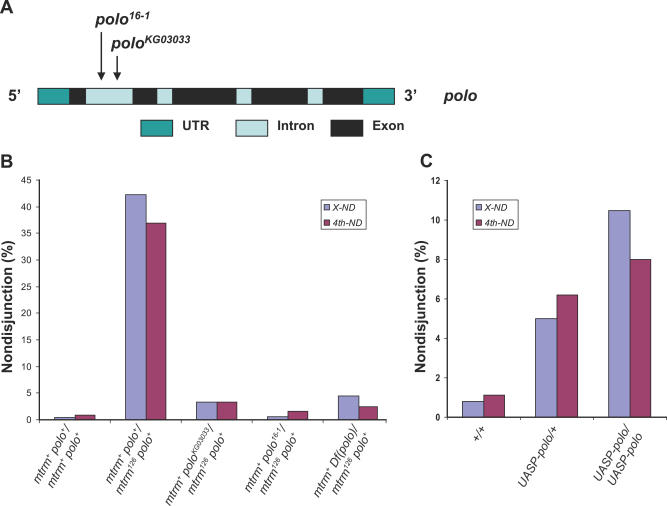
Reducing the Dose of *polo^+^* Suppresses *mtrm* Defects, and Increasing the Dose of *polo^+^* Partially Mimics the Effects of *mtrm* (A) Schematic diagram of the *polo* gene (black boxes depict the five exons) indicating the insertions sites for the two *polo* alleles (*polo^16–1^* and *polo^KG03033^*). (B) Summary of the genetic interaction of *mtrm* and *polo* mutants as examined by assaying the frequency of nondisjunction of *X* and *4* ^th^ chromosomes. As shown by Harris et al. [9], *mtrm/+* heterozygotes display high levels of nondisjunction for both achiasmate *X* and *4* ^th^ chromosomes (42% and 37%, respectively) when compared to *mtrm^+^/mtrm^+^* females. However, simultaneously reducing the dose of *polo,* as a result of heterozygosity for either the two P element insertion site mutants or a deficiency that uncovers *polo* (*Df(3L)rdgC-co2*) suppresses the meiotic phenotype of *mtrm/+* heterozygotes. (C) Expression of the *UASP-polo^+^* transgene in *mtrm^+^/mtrm^+^* females results in a dose-dependent increase in the frequency of achiasmate nondisjunction for both the *X* and the *4* ^th^ chromosomes. However, two weaker alleles of polo, *polo^01673^* and *polo^1^*, showed little or no suppression of the segregational defect (unpublished data). The *polo^1^* mutant, which is the weakest of the known *polo* mutants (it is viable over a deletion) is the result of a point mutation at base pair 725, V242E, in the kinase domain. Although *polo^01673^* is recessive lethal, it must retain some degree of function because it complements at least one other hypomorphic allele of *polo*, *polo^x8^*. The results indicate that reduction of *polo^+^* dosage rescues *mtrm* defects and the suppressive effect of a given *polo* mutant correlates with the severity in the reduction of Polo function.

*mtrm/+* heterozygotes display a substantial defect in the processes that ensure the segregation of achiasmate homologs. We show here that these meiotic defects are strongly suppressed by simultaneous heterozygosity for strong loss-of-function alleles of *polo*. (Our impetus for searching for a genetic interaction between *mtrm* and *polo* came from the finding that the mutants in the *mei-S332* gene were partially suppressed by *polo* mutants [12].) We measure meiotic mis-segregation by assaying *X* and *4* ^th^ chromosomal nondisjunction in females of the genotype *FM7/X* where *FM7* is a balancer chromosome that fully suppresses *X* chromosomal exchange. (The *4* ^th^ chromosome is obligately achiasmate.) As shown in Figure 3B, *FM7/X*; *mtrm/+* females typically show frequencies of *X* and *4* ^th^ chromosome nondisjunction in the range of 35%–45%, which is more than 100-fold above control values.

However, *FM7/X*; *mtrm^126^/+* females that were simultaneously heterozygous for either a deficiency (*Df(3L)rdgC-co2*) that uncovers *polo* or for either of two strong alleles of *polo*, *polo^KG03033^* and *polo^16–1^* (Figure 3A) displayed greatly reduced levels of meiotic nondisjunction (Figure 3B). The fact that the *polo^KG03033^* mutation is due to a P element insertion allowed us to demonstrate that the observed interaction with *mtrm* was indeed a direct consequence of a reduction in *polo* activity. Two precise excisions of this insertion were generated, and neither was able to suppress the nondisjunctional effects observed in *mtrm/+* heterozygotes (unpublished data). We also demonstrated that the *polo^KG03033^* allele was able to suppress the meiotic defects generated by heterozygosity for *mtrm^exc13^*, an independently isolated allele of *mtrm* (unpublished data).

Heterozygosity for these same loss-of-function alleles of *polo* has no detectable effect on meiotic chromosome segregation in *mtrm^+^/mtrm^+^* females. In females of the genotypes *FM7/X*; *polo^KG03033^/+* or *FM7/X*; *polo^16–1^/+*, the observed levels of nondisjunction for the *X* chromosome were 0.2% and 0.4%, respectively. Similarly, the observed levels of nondisjunction for the *4* ^th^ chromosome were 0.6% and 0.5% respectively (*n* = 1,109 for *FM7/X*; *polo^KG03033^ /+* and *n* = 1,226 for *FM7/X*; *polo^16–1^/+* females). These data alone are consistent with either a hypothesis in which Mtrm acts to inhibit Polo, excess Polo creates a meiotic defect or a scenario in which Polo inhibits Mtrm, and the absence of sufficient Mtrm creates the defect. However, as we will show below, our additional data support the model whereby Mtrm inhibits Polo.

### Increasing the Dose of *polo^+^* Partially Mimics the Effects of *mtrm* and Enhances the Defects Observed in *mtrm/+* Heterozygotes

If reducing the quantity of Polo suppresses the meiotic defects observed in *mtrm/+* females, then over-expression of Polo alone should mimic the effects of reducing the dosage of *mtrm^+^* (i.e., we should see a chromosome segregation defect solely in the presence of increased dosage of *polo^+^*, even in *mtrm^+^/mtrm^+^* oocytes). To test this hypothesis, we analyzed *FM7/X* females carrying two doses of a *UASP-polo^+^* transgene construct driven by the *nanos-GAL4* driver. As shown in Figure 3C, expression of the *UASP-polo^+^* transgene construct results in a dosage-dependent increase in the frequency of achiasmate nondisjunction for both the *X* and the *4* ^th^ chromosomes. Similar observations were made using chromosomal duplications that carry two copies of *polo^+^* (Adelaide Carpenter, personal communication). Moreover, increasing the dose of Polo in females heterozygous for *mtrm^126^* resulted in severe meiotic defects. Females carrying a single copy of the *UASP-polo^+^* transgene and which were also heterozygous for *mtrm^126^* were virtually sterile (unpublished data). Thus, increasing the dosage of Polo enhances the defect observed in *mtrm/+* heterozygotes by inducing sterility.

The genetic interaction between Mtrm and Polo during oogenesis is paralleled by their patterns of expression. Mtrm reaches its maximum level of expression from the end of stage 10 onward, filling the oocyte during stages 11–12, and then diminishes at stage 13. Analysis of Polo expression using an antibody to Polo [13,14] and wild-type oocytes revealed that Polo is present in the oocyte at low levels (except in the germarium) until stages 11 or 12 and then rapidly fills the oocyte cytoplasm from stages 12–13 onward (Figure S1). Taken together, these data support a model in which the presence of Mtrm inhibits Polo in the early stages of expression, while permitting the function of Polo at stage 13, when Mtrm is degraded. Data directly demonstrating that assertion are provided below.

### Mtrm and Polo Physically Interact In Vivo

**Figure 4:**
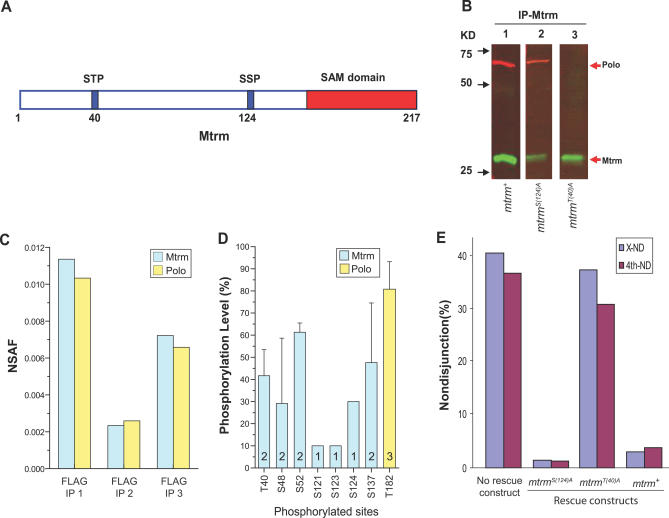
Mtrm Physically Interacts with Polo with a Stoichiometry of Approximately 1:1 (A) Schematic depiction of the Mtrm protein. Mtrm has two potential PBD-binding sites, STP and SSP, with the central serine/threonine residue at 40 and 124, respectively, and a SAM domain at the C terminus. Two independent transgenes expressing mutated PBD-binding sites were generated: Mtrm^T(40)A^, which disrupts the STP site and Mtrm^S(124)A^, which disrupts the SSP site. (B) Mtrm and Polo physically interact as shown by coimmunoprecipitation experiments. An antibody to Mtrm precipitates Polo from wild-type ovary extracts (lane 1). Expression of the mutated PBD binding site constructs in a *mtrm* null background reveals that Mtrm^S(124)A^ does not ablate the Mtrm-Polo interaction (lane 2); however, Mtrm^T(40)A^ failed to bind Polo (lane 3), indicating that the STP motif is critical for the Mtrm-Polo interaction. (C) Three independent affinity purifications from ovarian extracts expressing a C-terminally 3× FLAG-tagged Mtrm were used for the MudPIT mass spectrometry assay. Among the reproducible and significant (*p-*value < 0.001) proteins identified in all three analyses, Polo was detected by multiple peptides and stands out as the only protein recovered at levels similar to those of Mtrm, as estimated by NSAF. (D) Phosphorylated sites detected in Mtrm (blue bars) and Polo (yellow bar) are shown. Modification levels are estimated based on local spectral count and averaged across the three immunoprecipitations. The underlined numbers in each bar represent the number of times (out of three) the residues were found modified. (E) The STP site required for Polo binding is also required for Mtrm function. As noted above, *FM7/X*; *mtrm/+* heterozygotes display approximately 40% *X* nondisjunction (ND) and 37% *4* ^th^ nondisjunction. Although the Mtrm^S(124)A^ protein was able to rescue the meiotic defect (3.6% *X* and 4.4% *4* ^th^ ND), the Mtrm^T(40)A^ protein displayed similar levels of nondisjunction as *mtrm/+* heterozygotes, indicating that the STP motif is critical for Mtrm function. The finding that only the STP site is required for both Mtrm function and the binding of Mtrm to Polo is consistent with the observation that only the STP motif is conserved across all twelve sequenced *Drosophila* genomes, whereas the SSP motif is conserved only within the six species that belong to the *D. melanogaster–D. ananassae* clade.

A large scale yeast two-hybrid screen identified Mtrm as a candidate interactor with Polo [10] and showed that Mtrm carries two putative PBD binding sites: STP and SSP (Figure 4A). To confirm that Mtrm interacts with Polo physically in vivo, we performed coimmunoprecipitation experiments on wild-type ovary extracts using a polyclonal antibody to Mtrm. As shown in lane 1 of Figure 4B, the antibody to Mtrm also precipitated Polo.

We used two separate approaches to confirm the interaction between Polo and Mtrm. In the first experiment, we used ovary extracts from females expressing a *Green Fluorescent Protein* (*GFP*)*-polo* transgene [13] and performed the coimmunoprecipitation using an antibody to GFP. In the second experiment, we used ovary extracts from wild-type females and performed the coimmunoprecipitation using a monoclonal antibody to Polo [14]. In both experiments, we were able to show that Mtrm coimmunoprecipitated with Polo (Figure S2).

In addition, MudPIT mass spectrometry reveals that Mtrm and Polo interact in oocytes with a stoichiometry of approximately 1:1**.** We analyzed three independent affinity purifications from ovarian extracts expressing a C-terminally 3× FLAG-tagged Mtrm, and we used MudPIT mass spectrometry [15] to identify interacting proteins. We then compared the identified proteins to those detected in five control FLAG immunoprecipitations from control (*w^1118^*) flies. Among the proteins that showed reproducible and significant *p-*values (*p* < 0.001) identified in all three analyses, Polo was detected by multiple peptides and stands out as the only protein recovered at levels similar to those of Mtrm, as estimated by normalized spectral abundance factor (NSAF) counts [16,17]. Although the NSAF values for Mtrm and Polo vary across the three biological replicates analyzed (Figure 4C), the ratio between the two proteins remains constant with an average of 0.96 ± 0.11, suggesting one Mtrm molecule binds to one molecule of Polo.

Thus, three lines of evidence demonstrate that Mtrm physically interacts with Polo: the yeast two-hybrid work [10], our coimmunoprecipitation studies, and our MudPIT mass spectrometry experiments presented in this section. The observation of strong genetic interactions between mutants in these two genes (Figure 3) demonstrates a functional significance to this interaction.

### Mutation of the First PBD Binding Site of Mtrm Both Prevents Its Ability to Interact with Polo and Ablates Mtrm Function

Polo interacts with target proteins via the interaction of its PBD and the sequences STP or SSP on the target protein. In both of these PBD-binding sites, the center residues (threonine or serine) are phosphorylated to facilitate Polo binding [18–20]. Mtrm carries two potential PBD-binding sites: STP with the central threonine at residue 40 and SSP with the central serine at residue 124 (Figure 4A). To determine whether the interaction between Mtrm and Polo is mediated through the interaction of the Polo PBD with either or both of these two potential PBD-binding sites, we created *UASP*-driven transgenes that carried mutations in either or both of the STP or SSP motifs. In each case, we mutated the central residue of the PBD-binding sites on Mtrm to the nonphosphorylateable residue alanine. These mutants are denoted as *mtrm^T(40)A^*, which disrupts the STP motif, and *mtrm^S(124)A^*, which disrupts the SSP motif. Each of these mutant constructs was expressed under the control of the *nanos-GAL4* driver in a *mtrm* null background to insure that they were the only source of Mtrm protein in the oocytes. Coimmunoprecipitation experiments using antibodies to Mtrm revealed that Mtrm^S(124)A^ protein still interacted with Polo (Figure 4B). However, Mtrm^T(40)A^ failed to bind to Polo (Figure 4B), indicating that the STP residues define a motif that is critical for the Mtrm–Polo interaction. Mutation of both PBD sites also resulted in a version of Mtrm that did not interact with Polo (unpublished data).

Because the interaction of Polo with target proteins via its PBD requires the phosphorylation of the center residues (threonine or serine) of the STP or SSP motifs [18–20], we searched the MS/MS dataset for phosphorylated peptides derived from Mtrm or Polo. For each of the detected sites, we estimated the levels of modification by dividing the number of spectra matching a particular phosphopeptide by the total spectral count for this peptide (Figure 4D). We were able to detect phosphorylation on both T40 and S124; although, in agreement with the second PBD not being the primary binding site, S124 phosphorylation was found less reproducibly. In addition, Mtrm S48, S52, and S137 were found phosphorylated at reproducibly high levels in two out of three experiments. We also observed that Polo T182 was detected as phosphorylated at high levels (over 80%) in all three immunoprecipitations, indicating that those Polo proteins that are bound to Mtrm were fully activated [21].

Not only is the STP motif important for Polo binding, but it is also required for proper Mtrm function (Figure 4E). We assayed the frequency of nondisjunction in females expressing either the *mtrm^S(124)A^* or the *mtrm^T(40)A^* construct in the germlines of *FM7/X*; *mtrm/+* heterozygotes. Although the *mtrm^S(124)A^* construct was able to rescue the meiotic defects seen in *mtrm/+* heterozygotes, the *mtrm^T(40)A^* construct failed to rescue the *mtrm* defect and maintained the high nondisjunction frequency seen in *FM7/X*; *mtrm/+* heterozygotes. A similar failure to rescue was observed using a double mutant construct that carried both the *mtrm^S(124)A^* and the *mtrm^T(40)A^* mutations (unpublished data). Based on these observations, we conclude that the STP site is critical for Mtrm function and the T(40)A mutation ablates Mtrm function as a direct consequence of a failure to interact with Polo.

### Mtrm Functions as an Inhibitor of Polo

In the previous sections, we presented three separate lines of evidence that Mtrm acts to inhibit Polo function, and not vice versa. First, effects of heterozygosity for *mtrm* can be suppressed by a corresponding reduction in the dose of *polo^+^*. Second, we observed that the phenotype created by reducing the dose of *mtrm^+^* can be mimicked by increasing the dose of Polo. Third, and most importantly, the observation that mutating the STP Polo binding site by a conservative amino acid replacement (STP → SAP) ablates Mtrm function argues strongly that Mtrm functions as an inhibitor of Polo. Were it the case that Polo inhibits Mtrm, one would expect loss of the Polo interacting site to produce a hyperfunctional Mtrm, not a nonfunctional protein.

### As Either a Heterozygote or a Homozygote, *mtrm* Causes Precocious NEB

The early stages of meiosis appear normal in both *mtrm/+* and *mtrm/mtrm* oocytes. The germarium and early stages appear morphologically normal and, at least in *mtrm/+* oocytes, both recombination and SC assembly are indistinguishable from normal ([9] and unpublished data). However, following stage 11 (the period during which Mtrm is maximally expressed), we observed multiple defects in oocyte maturation in both *mtrm/+* and *mtrm/mtrm* oocytes. Most critically, we show that a loss-of-function allele of *mtrm* induces precocious NEB in a dosage-dependent manner.

**Figure 5:**
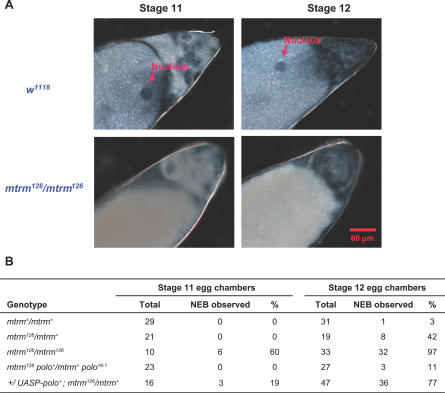
*mtrm* Causes Precocious NEB (A) Representative examples of NEB in stage 11 and 12 egg chambers for wild-type (*w^1118^*) and *mtrm^126^* homozygotes. NEB in wild-type oocytes occurs at stage 13. The nucleus is still present (seen as a dark mass by phase contrast microscopy) at stage 11 and stage 12 in wild-type. *mtrm* homozygotes show precocious NEB (absence of the dark mass) that can occur prior to stage 11. Scale, 60 μm. (B) Summary of NEB in stage 11 and stage 12 egg chambers for wild-type (*w^1118^*), *mtrm* heterozygotes (*mtrm^126^*/*+*), *mtrm* homozygotes (*mtrm^126^*/ *mtrm^126^*), double heterozygotes for both *mtrm*, *polo* (*mtrm^126^ +*/*+ polo^16–1^*), and over-expression of Polo in *mtrm* heterozygotes (*+/UASP-polo; mtrm^126^/+*).

In wild-type oocytes, NEB usually does not occur until stage 13; only a single case of NEB at stage 12 was observed among the 61 stage 11 and 12 wild-type oocytes examined (Figure 5). However, in *mtrm^126^*/+ heterozygotes, more than a third of stage 12 egg chambers exhibited NEB. To ensure that the precocious NEB defect is the consequence of reducing the copy number of *mtrm^+^*, we repeated these experiments using females that are heterozygous for an independently isolated allele of *mtrm*; *mtrm^exc13^*. These females also displayed precocious NEB at stage 12 (data not shown). As is the case for the chromosome segregation defects observed in *mtrm/+* oocytes, the precocious NEB that is seen in *mtrm^126^*/+ heterozygotes is strongly suppressed by simultaneous heterozygosity for a loss-of-function allele of *polo* (Figure 5B), suggesting that the timing of NEB is determined by the relative abundances of Mtrm and Polo. This conclusion is further strengthened by the observation that overexpression of Polo (using a *UASP-polo^+^* transgene driven by the *nanos-GAL4* driver) increases the frequency of precocious NEB in *mtrm^126^/+* heterozygotes by nearly 2-fold (from 42% to 77%).

The extent of the precocious NEB defect is even more evident in *mtrm^126^* homozygotes. As shown in Figure 5, NEB had already occurred in 32 out of 33 stage 12 oocytes examined and in six of ten stage 11 oocytes examined. Thus, the loss of Mtrm causes precocious NEB in a dosage-dependent fashion. Taken together, these data argue that the presence of Mtrm prevents Polo from inducing NEB until stage 13 (see Discussion), and that a reduction or absence of available Mtrm allows the Polo that is synthesized during stages 11 and 12 to initiate NEB.

**Figure 6:**
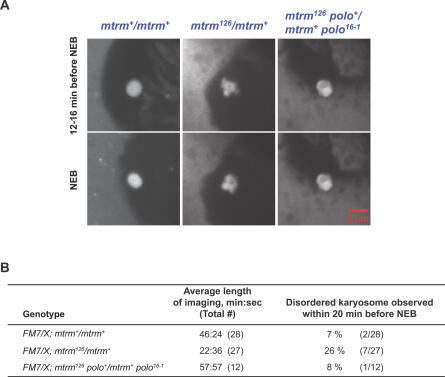
*mtrm* Is Defective in Karyosome Maturation before NEB The karyosomes in stage 11–12 oocytes, which have a nuclear envelope, were imaged after the injection of Oli-green and Rhodamine-tubulin until NEB. NEB was defined as the time when the nuclear envelope seems ruffled and the Rhodamine-tubulin enters the nucleus. (A) Representative examples of karyosomes 12–16 min before and at NEB are shown for wild type, *mtrm^126^/mtrm^+^*, and *mtrm^126^ polo^+^/mtrm^+^ polo^16–1^* with achiasmate *X* chromosomes (*FM7/X*). Wild type displays a circular karyosome with a smooth outline for 12–16 min before NEB, whereas *mtrm^126^/mtrm^+^* oocytes bear scabrous or bi-lobed karyosomes. The disordered morphology of karyosomes in *mtrm^126^/mtrm^+^* oocytes was suppressed by simultaneously reducing the dose of *polo*. Scale, 5 μm. (B) Summary of karyosome morphology during the 20 min before NEB.

The precocious breakdown of the nuclear envelope at stages 11 to 12 is important because the karyosome undergoes dramatic changes in structure during this period [2]. As noted above, in stages 9–10, the karyosome expands to the point that individual chromosomes can be detected [22–24]. These chromosomes recondense into a compact karyosome during stages 11–12, the exact time at which a reduction in the level of Mtrm causes precocious NEB. Thus, the early NEB events promoted by heterozygosity for *mtrm* might be expected to result in the release of incompletely condensed or disordered karyosomes. To test this hypothesis, we examined karyosome morphology during the 20 min that preceded NEB in wild-type*, mtrm^126^/mtrm^+^*, and *mtrm^126^ polo^+^/mtrm^+^ polo^16–1^* oocytes. As shown in Figure 6, only two out of 28 (7%) wild-type oocytes with incompletely compacted or disordered karyosomes were observed. However, 7 out of 27 (26%) *mtrm^126^/mtrm^+^* oocytes displayed a disordered karyosome, an effect that was largely suppressed (to 8%) by simultaneous heterozygosity for *polo^16–1^* (Figure 6). These data support the view that the precocious NEB that is induced by lowering the level of Mtrm results in the release of improperly formed karyosomes into the cytoplasm and are again consistent with the possibility that Mtrm inhibits meiotic progression through its effects on Polo.

### Mtrm Is Also Required to Maintain Karyosome Structure after NEB

**Figure 7:**
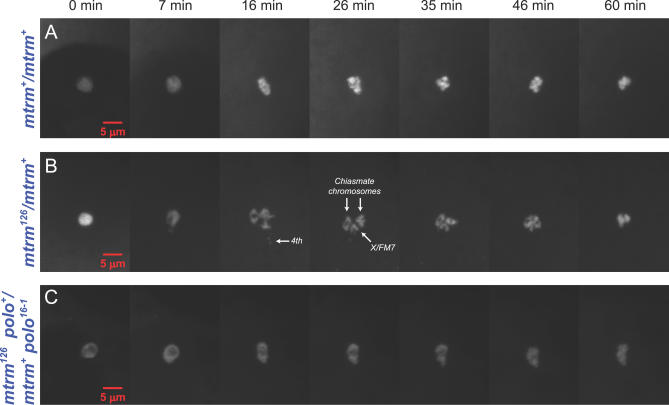
*mtrm* Causes the Individualization of Bivalents after NEB Stage 12 oocytes were injected with Oli-green to visualize karyosomes. After this injection, we analyzed the change in karyosome structure during NEB using live imaging. Time frames from NEB (time 0) are shown for: (A) *FM7/X; mtrm^+^/mtrm^+^* for control; (B) *FM7/X; mtrm^126^/mtrm^+^*; and (C) *FM7/X; mtrm^126^ polo^+^/mtrm^+^ polo^16–1^* oocytes (see also Videos S1–S3). In control oocytes, the karyosome stays condensed after NEB and then becomes elongated at about 13 min, presumably as a consequence of the chromosomes establishing proper centromere co-orientation. As noted in the text, almost all control oocytes (8/9) exhibited a karyosome in which chromosomes are tightly associated. In the remaining case, three bivalents could be distinguished but were still physically associated. However, in *FM7/X; mtrm^126^/mtrm^+^* oocytes, the *4* ^th^ chromosomes are separated from a single mass of chromatin at 6–8 min after NEB, and then *X, 2^nd^* and *3^rd^* chromosomes start to spread out. At approximately 16 min after NEB, they are individualized into three obvious and fully separate bivalents. Individualized chromosomes begin to re-condense around 46 min and form a single mass. Indeed, the majority (11/15) of those oocytes that underwent bivalent individualization eventually formed bipolar spindles with the chiasmate chromosomes properly balanced on the metaphase plate (see also Figure 4 of Harris et al. [9]). Thus the karyosome maintenance defect induced by heterozygosity for *mtrm* does not permanently impair the progression of prometaphase. Finally, the karyosome maintenance defect induced by heterozygosity for *mtrm* was suppressed by reducing the dosage of the *polo^+^* gene. As noted in the text, 10 of the 13 *FM7/X; mtrm^126^ polo^+^/mtrm^+^ polo^16–1^* oocytes maintained the karyosome as a single mass throughout the process of spindle assembly. The three remaining cases may be described as follows: (1) the karyosome dissolved into three clearly distinguishable bivalents, but this oocyte did not ever succeed in forming a bipolar spindle; (2) the three major bivalents could be distinguished but did not physically separate; and (3) in an oocyte that may have been leaking or damaged, the bivalents individualized at about 8 min after the initiation of spindle assembly, but their morphology was abnormally stretched and thread-like. 7 min later, these chromosomes began to fragment into much smaller pieces, which led to the assembly of a spindle with at least five and possibly more poles. It seems likely that this case reflects simply the fragility of the karyosome, even in *polo^16–1^/polo^+^* suppressed oocytes, rather than the defect observed in *FM7/X; mtrm^126^/mtrm* oocytes that are wild type for *polo*.

The karyosome plays a critical role in directing the formation of the acentriolar spindle in *Drosophila* oocytes. In 8 out of 9 (89%) wild-type oocytes, the karyosome remains associated even after NEB; it is then surrounded by microtubules and forms a bipolar meiotic spindle (Figure 7 and Video S1). At metaphase I, chiasmate chromosomes are still condensed into a single mass at the metaphase plate in a tapered bipolar spindle [25–28].

However, in *FM7/X*; *mtrm^126^/mtrm^+^* oocytes, the karyosome usually dissolved within 10–20 min after NEB, and the individual bivalents became clearly visible (Figure 7 and Video S2). In 15 out of 17 (88%) *FM7/X; mtrm^126^/ mtrm^+^* oocytes examined, the chromosomes were individualized during spindle assembly. Indeed, in 14 of these examinations, all three pairs of major chromosomes were physically separated at some point during the time course of imaging (in the remaining case, the three bivalents could be distinguished but were still physically associated). As discussed in the legend to Figure 7, despite this dissociation into individual bivalents, in most oocytes the chromosomes are capable of reaggregating into a single mass and eventually forming a bipolar spindle.

A striking example where all four chromosome pairs can be clearly distinguished is the image taken 26 min after NEB for *FM7/X*; *mtrm^126^/mtrm^+^* oocytes (Figure 7). In those oocytes in which bivalent individualization was observed, the two major autosomes appeared to be held together by at least two chiasmata (one on each arm), suggesting that sister-chromatid cohesion along the euchromatic arms of these chromosomes still persists. The two *X* chromosomes remain physically associated, despite the lack of chiasmata, presumably as a consequence of the maintenance of heterochromatic pairing [29,30].

Because the nondisjunction of achiasmate chromosomes observed in *mtrm^126^/mtrm^+^* heterozygotes was suppressed by heterozygosity for loss-of-function alleles of *polo*, we next tested whether a *polo* mutation could also suppress this karyosome maintenance defect. As shown in Figure 7 and Video S3, bivalent individualization was only observed in three out of 13 (23%) of *FM7/X; mtrm^126^ polo^+^/mtrm^+^ polo^16–1^* oocytes, and thus 77% of the oocytes maintained the karyosome as a single mass throughout the process of spindle assembly. These data are consistent with the genetic data presented above: reducing the dose of *polo^+^* strongly suppresses the deleterious effects of heterozygosity for *mtrm*.

### The Defects in Karyosome Maintenance Are Followed by Defective Co-Orientation of Achiasmate Centromeres on the Meiotic Spindle

**Figure 8:**
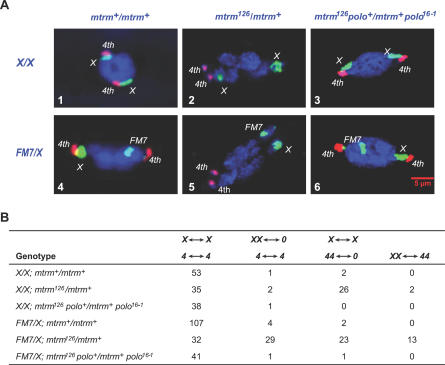
Heterozygosity for *mtrm^126^* Impairs the Proper Co-Orientation of Achiasmate Centromeres during Prometaphase (A) FISH analysis using probes homologous to the *X* and *4* ^th^ chromosomal heterochromatin [29] were used to assay centromere co-orientation during meiotic prometaphase. In *mtrm^+^*/ *mtrm^+^* oocytes carrying either chiasmate *X* chromosomes (*XX* females) or achiasmate *X* chromosomes (*FM7/X* females), the centromeres of both the *X* and the *4* ^th^ are virtually always oriented toward opposite poles (see panels 1 and 4 and (B)). However, in *mtrm/+* heterozygotes, the centromeres of achiasmate bivalents are often oriented towards the same pole (see panels 2 and 5 and (B)). In double heterozygotes for both *mtrm* and *polo*, these defects in achiasmate chromosome centromere co-orientation are greatly suppressed (panels 3 and 6). (B) Quantitative summary of centromere co-orientation patterns for the various genotypes studied. Although heterozygosity for *mtrm^126^* has a dramatic effect on *4* ^th^ chromosome centromere malorientation in both *XX* and *FM7/X* females, there is little effect on *X* chromosome segregation in *XX* oocytes when compared with the dramatic effect observed in *FM7/X* females. This is to be expected based on the genetic studies of Harris et al. [9], who observed that only achiasmate bivalents nondisjoin in *mtrm/+* females.

Because the karyosomes of *mtrm/+* females were poorly formed before NEB and are usually transiently dissolved to individual bivalents shortly after NEB (see above), we also examined centromere co-orientation on bipolar prometaphase spindles using FISH probes (see Materials and Methods) directed against the *X* and *4* ^th^ chromosomes (Figure 8) in both wild-type and *mtrm/+ oocytes*.

In wild-type oocytes, the vast majority of most *X* and *4* ^th^ chromosome centromeres co-oriented properly (Figure 8). The frequencies of abnormal centromere co-orientation in oocytes with chiasmate *X* chromosomes (*XX*) were only 2% for the *X* chromosome and 4% for the *4* ^th^ chromosome. In *FM7/X* females, where *X* chromosomal crossingover is blocked, the frequencies of abnormal co-orientation were still quite low (4% for the *X* chromosome and 2% for the *4* ^th^). However, co-orientation of achiasmate centromeres was often aberrant in *mtrm/+* heterozygotes, such that the centromeres of both homologs were often oriented toward the same pole (Figure 8A). In these cases, the two homologs also occupied different arcs of the meiotic spindle, a feature that is rarely, if ever, observed in wild-type oocytes. In chiasmate *X* females, 43% of observed oocyte nuclei displayed an aberrant co-orientation of *4* ^th^ chromosome centromeres, and 6% of these oocytes displayed aberrant *X* centromere co-orientations (Figure 8B); these oocytes likely reflect the 8%–10% of oocytes that fail to undergo crossingover even in females bearing structurally normal *X* chromosomes. The defect in *4* ^th^ chromosome centromere co-orientation was fully suppressed by simultaneous heterozygosity for *polo^16–1^* (Figure 8A and 8B).

As expected, due to the suppression of *X* chromosomal crossingover in *FM7/X* females, *mtrm/+* heterozygotes displayed frequent abnormal centromere co-orientation for both *X* and *4* ^th^ chromosomes, i.e., 43% for *X* chromosomes and 37% for *4* ^th^ chromosomes (Figure 8B). These results indicate that the *mtrm* heterozygotes display an obvious defect in centromere co-orientation. However, once again, both the defect in *X* and *4* ^th^ chromosome centromere co-orientation was fully suppressed by simultaneous heterozygosity for *polo^16–1^*. Thus, as was the case with the previously considered defects, the deleterious effects of reducing the amount of available Mtrm can be suppressed by a simultaneous reduction in the amount of Polo.

## Discussion

The data presented above argue that Mtrm serves to inactivate newly synthesized Polo during the period of meiotic progression that precedes NEB. An excess of functional (unbound) Polo, produced by reducing the amount of available Mtrm, causes the early onset of NEB. This early entry into prometaphase releases an immature karyosome into the cytoplasm, which then fails to properly align the centromeres of achiasmate chromosomes on the prometaphase spindle. These observations raise a number of questions ranging from the role of Polo in mediating the G2/M transition in oogenesis to the role of the karyosome structure in facilitating the proper segregation of achiasmate chromosomes.

### Polo Plays a Critical Role in Initiating the G2/M Transition in Oogenesis by Regulating Cdc25

The trigger for the G2/M transition is activation of Cdk1 by Cdc25 (reviewed in [31]), and multiple lines of evidence suggest that Polo can activate Cdc25 [32]. First, in Caenorhabditis elegans, RNAi experiments demonstrate that ablation of Polo prevents NEB [33]. Second, the *Xenopus* Polo homolog Plx1 is activated in vivo during oocyte maturation with the same kinetics as Cdc25. Additionally, microinjection of Plx1 accelerates the activation of both Cdc25 and cyclinB-Cdk1 [34]. Moreover, microinjection of either an antibody to Plx1 or kinase-dead mutant of Plx1 inhibited the activation of Cdc25 and its target cyclinB-Cdk1. A later study by Qian et al. demonstrated that injection of a constitutively active form of Plx1 accelerated Cdc25 activation [35]. As pointed out by these authors, these studies support “the concept that Plx1 is the ‘trigger' kinase for the activation of Cdc25 during the G2/M transition.” Finally, a small molecule inhibitor of Polo kinase (BI 2536) also results in extension of prophase [36]. These data are consistent with the view that the presence of functional (unbound) Polo plays a critical role in ending the extended G2 that is characteristic of oogenesis in most animals. We should note by Riparbelli et al. [37] that the careful study of female meiosis in *polo^1^* homozygotes failed to observe a defect in the timing of NEB. However, as discussed in the legend to Figure 3, *polo^1^*, a missense mutant that is viable even over some deficiencies and does not suppress *mtrm*, is the weakest of the known *polo* mutants, and it is thus reasonable that no defect was observed.

**Figure 9:**
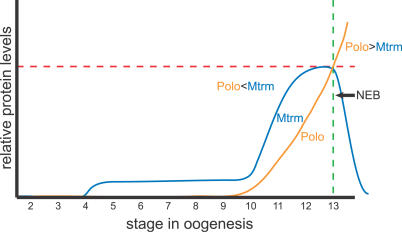
A Model for the Control of NEB by Mtrm-Induced Inhibition of Polo According to this model, in wild-type *Drosophila* oocytes, the excess of Mtrm inhibits those Polo proteins that are deposited in the oocyte during stages 11 to 12. However, by stage 13, an excess of Polo exceeds the available amount of inhibitory Mtrm proteins. This unencumbered Polo then serves to activate Cdc25, initiating the chain of events that lead to NEB and the initiation of prometaphase. In the absence of a sufficient amount of Mtrm, an excess of functional Polo causes the precocious activation of Cdc25 and thus an early G2/M transition. Based on this model, one can visualize that decreasing the dose of Mtrm or increasing the dosage of Polo will hasten NEB, while simultaneous reduction in the level of both proteins will normalize the timing of NEB.

In light of these data, it is tempting to suggest that in wild-type *Drosophila* oocytes, the large quantity of Mtrm deposited into the oocyte from stage 10 onward inhibits the Polo that is either newly synthesized or transported into the oocyte during stages 11–12. However, at stage 13, an excess of functional Polo is created when the number of Polo proteins exceeds the available amount of inhibitory Mtrm proteins. This unencumbered and thus functional Polo then serves to activate Cdc25, initiating the chain of events that leads to NEB and the initiation of prometaphase. In the absence of a sufficient amount of Mtrm, an excess of Polo causes the precocious activation of Cdc25, and thus an early G2/M transition. A model describing this hypothesis is presented in Figure 9. Based on this model, one can visualize that decreasing the dose of Mtrm or increasing the dose of Polo will hasten NEB, whereas simultaneous reduction in the dose of both proteins should allow for proper timing of NEB.

**Figure 10:**
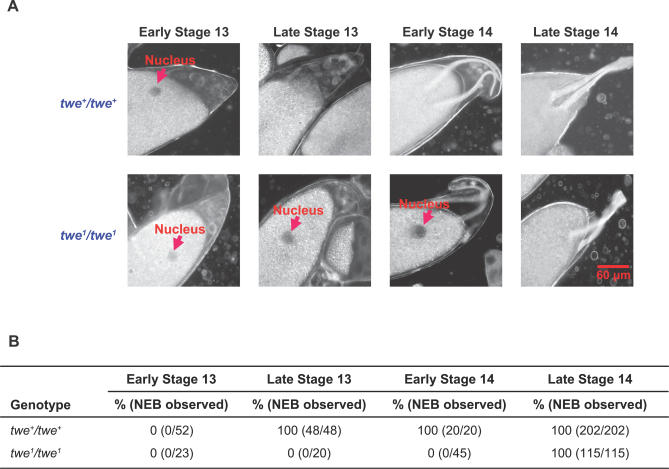
Mutants in the *Drosophila cdc25* Homolog *twine* Fail to Undergo NEB in Stage 13 (A) Representative examples of NEB in stage 13 and 14 egg chambers for wild-type (*w^1118^*) and *twine* (*twe^1^* ) homozygotes. The nucleus is present (seen as a dark mass by phase contrast microscopy) at early stage 13 but not at late stage 13 and stage 14 in wild type. *twe1* homozygotes show delayed NEB and that the nucleus is still present until early stage 14. Scale, 60 μm. (B) Summary of NEB in stage 13 and stage 14 egg chambers for wild-type (*w^1118^*) and *twe^1^* homozygotes (*twe^1^/ twe^1^*).

Two lines of evidence directly support a model in which Mtrm exerts its effect on Polo, with respect to preventing precocious NEB, by blocking the ability of Polo to activate Cdc25. First, as shown in Figure 10, mutants in the *Drosophila cdc25* homolog *twine* fail to undergo NEB in stage 13. In addition, heterozygosity for *twine* also decreases the frequency of precocious NEB in *mtrm^126^/+* heterozygotes from 42% (Figure 5) to less than 10% (7/72).

### How Might Mtrm Inhibit Polo?

Mtrm's first PBD binding site (T40) is required for its interaction with Polo. Mtrm T40 has to be first phosphorylated by a priming kinase, such as one of the Cdks or MAPKs, and was indeed detected as phosphorylated in the mass spectrometry dataset. The NetPhosK algorithm [38] predicts T40 to be a Cdk5 site, and the serines immediately distal to T40—S48 and S52—which were also detected as phosphorylated (Figure 4D), are sites for proline-directed kinases such as Cdk or MAPK sites as well. The other prominent phosphorylation event occurs at S137, which could be a Polo phosphorylation site since it falls within a Polo consensus (D/E-X-S/T-Ø-X-D/E). Although the combined sequence coverage for Mtrm was 44%, indicating that some phosphorylated sites might have been missed, Mtrm S137 is a suitable binding site for activated Polo, in agreement with the processive phosphorylation model [18]. At this point of our studies, Mtrm T40 priming kinase or the kinase responsible for Polo activating phosphorylation on T182 has not been identified.

The finding that Polo not only is able to bind to Mtrm in vivo in a 1:1 ratio, but also is fully phosphorylated on T182 in its activation loop [21] suggests a method by which Mtrm serves to inhibit Polo. In general, enzymes are usually not recovered from affinity purifications at levels similar to their targets. They do not form stable complexes, but rather form transient interactions with their substrates, which is how efficient catalysis is achieved. Here, Mtrm is able to sequester activated Polo away in a stable binary complex over a long period of time. It is only when this equilibrium is disturbed at the onset of stage 13 by the production of an excess of Polo (as suggested in Figure 9) or by degradation of Mtrm that Polo can be released. The molecular determinants of the Mtrm::Polo sequestration event are not clear, but it would be interesting to test whether the serines found phosphorylated in the vicinity of Mtrm PBD binding sites play a role in locking the binary complex into place.

### *mtrm* Exerts Its Effects on Achiasmate Nondisjunction Via a Cdc25-Independent Pathway

Our data demonstrate that a reduction in the levels of Mtrm results in the release of an incompletely compacted karyosome that rapidly dissolves into individual bivalents during the early stages of spindle formation. For chiasmate bivalents, this is apparently not a problem, because they still co-orient correctly (for example, the chiasmate *X* chromosomes shown in Figure 8 still achieve proper co-orientation in the vast majority of oocytes). However, the nonexchange bivalents frequently fail to co-orient properly, such that both homologs are oriented toward the same pole (but often occupy two different arcs of the spindle). This initial failure of proper co-orientation leads to high frequencies of nondisjunction as demonstrated by the genetic studies and analysis of metaphase I images presented in [9].

Although achiasmate homologs are properly co-oriented in wild-type oocytes [29,30] , we have noted previously such homologs can often vacillate between the poles such that two achiasmate homologs are often found on the same arc of the same half-spindle during mid to late prometaphase ([25] and unpublished data). These chromosomes are often observed to be physically associated. This situation is quite different from the defect observed in *mtrm/+* heterozygotes, where the homologs are neither physically associated nor on the same arc of the spindle.

It is tempting to suggest that the chromosome segregation defects we observe in *mtrm/+* heterozygotes are simply the result of precocious release of an incompletely re-compacted karyosome. According to this explanation, the defects observed in meiotic chromosome segregation are solely the consequence of premature NEB. (Implicit in this model is the assumption that it is the events that occur during karyosome re-compaction, at stages 11 and 12, that serve to initially bi-orient achiasmate chromosomes, and we do not have direct evidence to support such a hypothesis.)

Alternatively, Polo plays multiple roles in the meiotic process [7,8], and it is possible that the chromosome segregation defects we see represent effects of excess Polo that are un-related to the precocious breakdown of the nuclear envelope. Such a view is supported by two observations. First, as shown in Figure 7, the bivalent individualization observed after NEB in *mtrm*/+ oocytes does not disrupt *FM7-X* pairings. Second, although heterozygosity for *twine* in *mtrm^126^/+* heterozygotes suppresses the frequency of precocious NEB from 42% (Figure 5) to less than 10% (7/72), two alleles of *twine* tested (*twe^1^* and *twe^k08310^*) failed to suppress the levels of meiotic nondisjunction observed in *FM7/X; mtrm^126^/+* heterozygotes. These data suggest that the effects of excess Polo on nondisjunction may not be regulated *via* Cdc25/Twine, but rather by the effects of excess Polo on some other as-yet-unidentified Polo target. This suggests that the effects of Mtrm on the level of Polo might affect multiple Polo-related processes.

Support for such an idea that Mtrm can inhibit Polo-regulated proteins that are unrelated to NEB comes from the observation that the ectopic expression of *Drosophila* Mtrm in Schizosaccharomyces pombe blocks karyokinesis, producing long multi-septate cells with only one or two large nuclei ([39] and Bruce Edgar, personal communication). This phenotype is similar, if not identical to, that exhibited by mutants in the S. pombe Polo homolog *plo1* (Plo1), which fail in later stages of mitosis due to the role of Plo1 in activating the septation initiation network to trigger cytokinesis and cell division. However, Plo1 also plays a role in bipolar spindle assembly that might also be inhibited in the Mtrm expressing cells, but this function of Plo1 is less well understood.

Thus the possibility exists that the effect of *mtrm* mutants on meiotic chromosome segregation may well not be the direct consequence of early NEB, but rather may be due to the role of Polo in other meiotic activities, such as spindle formation or the combined effects of these defects with precocious NEB. Efforts to identify such processes and their components are underway in the lab.

Finally, we should note that while Mtrm is the first known protein that is able to inactivate Polo by physical interaction with Polo itself; there is certainly additional mechanisms of Polo regulation. For example, Archambault et al. [40] have described mutants in the gene that encodes Greatwall/Scant kinase, which have both late meiotic and mitotic defects. Although there is no evidence for a physical interaction between these two kinases, the authors speculate that the function of the Greatwall kinase serves to antagonize that of Polo. The *Scant* mutations create a hyperactive form of Greatwall, which might be expected to lower the dosage Polo, and thus perhaps partially suppress the defects observed in *mtrm/+* heterozygotes. Indeed, exactly such a suppressive effect has been observed in *Scant* homozygotes (however, this suppression is much weaker than that obtained by heterozygosity for loss of function alleles of *polo*).

### Summary

The data presented above demonstrate that Mtrm acts as a negative regulator of Polo during the later stages of G2 arrest during meiosis. Indeed, both the repression of Polo expression until stage 11 and the inactivation of newly synthesized Polo by Mtrm until stage 13 play critical roles in maintaining and properly terminating G2 arrest. Our data suggest a model in which the eventual activation of Cdc25 by an excess of Polo at stage 13 triggers NEB and entry into prometaphase. Although our data do shed some light on the mechanism by which Mtrm inhibits Polo, it is not entirely clear whether Polo's ability to phosphorylate targets other than Cdc25 might be blocked by Mtrm::Polo binding. These issues will need to be addressed in the future studies. Finally, we note that although small molecule inhibitors of Polo have been identified [36], Mtrm represents the first case of a protein inhibitor of Polo. It would be most exciting to identify functional orthologs of Mtrm outside of the genus *Drosophila*. Perhaps that might best be accomplished through a screen for oocyte-specific Polo-interacting proteins.

## Materials and Methods

### *Drosophila* stocks.

Throughout this study, a *w^1118^* stock served as our normal sequence *X* wild-type control, and for achiasmate *X* chromosome studies, *FM7/yw* was used as wild-type control. The *GFP-polo* stock was kindly provided by Adelaide Carpenter. The *nanos-GAL4* driver was used to express *UASP*-driven transgenes (see below) in the ovary. All *polo* mutants, the P element insertion mutant, and deficiencies related to *mtrm* were acquired from the Bloomington *Drosophila* Stock Center.

### Isolation and characterization of a null allele of *mtrm.*

A P-element insertion mutant, *KG08051*, causing a mutation in the *mtrm* gene and exhibiting high levels of nondisjunction for achiasmate chromosomes [9], was obtained from the Bloomington *Drosophila* Stock Center. Although Harris et al. [9] positioned the insertion site for this transposon 90 bp upstream of the first ATG in the *mtrm* coding sequence, resequencing indicates that the true insertion site is in fact 80 bp upstream of the first ATG in the *mtrm* coding sequence. *mtrm^126^* was generated by imprecise excision from the insertion of a null allele of *mtrm*. It is a deletion that removes 80 bp of 5′-UTR and 123 bp of coding sequence, deleting the first 41 amino acids (Figure 2A). Reverse-transcriptase (RT)-PCR, and Western blotting confirmed that *mtrm^126^* homozygotes had no transcripts and no protein expression (unpublished data). Like the original P element insertion mutant, *mtrm^126^* showed a dosage-sensitive effect on meiotic nondisjunction that was specific to achiasmate chromosomes and homozygous sterile females (homozygous males are fully fertile and meiotic segregation is normal in both *mtrm* heterozygotes and homozygotes).

### Construction of transgene plasmids.

To construct the *UASP-polo^+^* transgene, we amplified a 1.74-kb XhoI-XbaI *polo* fragment from reverse transcribed cDNA by PCR using the primers 5′-CTCGAGGATGGCCGCGAAGCCCGAGGATAAG-3′ and 5′-TCTAGATTATGTGAACATCTTCTCCAGCATTTTCC-3′. The *polo* fragment was cloned into the pBluescript to generate pBlue-*polo*-cDNA. Then, a *polo* fragment was obtained by digestion with KpnI and XbaI from pBlue-*polo*-cDNA and cloned into the *pUASP* vector [41] to produce *pUASP-polo^+^*. The *UASP-polo^+^* cassette in this plasmid was sequenced for confirmation. The transformation of the *pUASp-polo^+^* and other plasmids (see below), to generate transgenic flies, was conducted by Genetic Services in Boston, Massachusetts, United States.

To place the 3× FLAG downstream of *mtrm*, the PCR amplified 687-bp *mtrm*+1.5×-FLAG fragment was created using primer pKpnI-mtrm-5, 5′-GGGGTACCAAATGGAGAATTCTCGCACGCCCACGAACAAG-3′, and primer mtrm-3-FLAG (1.5×), 5′-GTCCTTGTAGTCCTTGTCATCGTCGTCCTTGTAGTCAAGAGTGTGGAGCACATCCATGATACGG-3′. Then the 687-bp *mtrm*+1.5× FLAG was amplified with the FLAG(3×)stop-Xbal primer, 5′-GCTCTAGATTACTTGTCATCGTCGTCCTTGTAGTCCTTGTCATCGTCGTCCTTGTAGTCCTTGTCATCGTCGTCCTTG- 3′, to produce the KpnI-XbaI *mtrm-Flag*(3×) fragment. The fragment was then cloned into the pUASP vector [41] to produce pUASP-*mtrm-flag(3x)*.

The Mtrm protein has two potential PBD binding sites: STP with the central threonine at residue 40 and SSP with the central serine at residue 124 (Figure 4A). To mutate the central residues to alanine in each motif, PCR assembly was used to make two separate codon changes in the *mtrm* gene, one at +118 from ACT to GCT to produce *mtrm^T(40)A^* and the other at +370 from CAG to CGC to produce *mtrm^S(124)A^*. To mutate the STP motif, primer pmtrm-mut-ATG: 5′-CGGGGTACCAAAAGATGGAGAATTCTCGCACGCCCACGAACAAGAC-3′ and primer pmtrm-STPre: 5′-GAGATTGGGCGAACGGAAGTTGCCAAAGATCGGAGCAGAGCATCGCACGTTGGAGGTGTTCACCTTCAG-3′ were used to amplify a 150-bp fragment for 5′-terminus of *mtrm.* The rest of *mtrm* was amplified with primers pmtrm-STP: 5′-CTGAAGGTGAACACCTCCAACGTGCGATGCTCTGCTCCGATCTTTGGCAACTTCCGTTCGCCCAATCTC-3′, and pmtrm-mut-TAA: 5′-GCTCTAGATTAAAGAGTG TGGAGCACATCCATGATACGCTTGC-3′ to produce a 520-bp fragment. The 150-bp and 520-bp fragments were combined in equal amounts and amplified by PCR to assemble the full-length KpnI-XbaI *mtrm^T(40)A^* gene introducing a point mutation. The KpnI-XbaI *mtrm^T(40)A^* was cloned in to pUASP to generate pUASP- *mtrm^T(40)A^*. After confirmation by sequencing, the plasmid was used for genetic transformation.

To construct the *mtrm^S(124)A^* transgene, primer pmtrm-mut-ATG and primer pmtrm-SSPre: 5′-GGTCTCCATATTCGAGTCATCCGAACAGGTATCCGGGGCGCTGCAGCTCT-3′ were used to amplify a 420-bp fragment of the 5′ terminus of *mtrm*. The 3′ terminus of *mtrm* was amplified by using primer pmtrm-SSP: 5′-AGAGCTGCAGCGCCCCGGATACCTGTTCGGATGACTCGAATATGGAGACC-3′and primer pmtrm-mut-TAA to produce a 300-bp fragment. The two fragments in equal molar amounts were amplified by PCR to assemble a full-length KpnI-XbaI *mtrm^S(124)A^* gene with a point mutation introduced. The KpnI-XbaI *mtrm^S(124)A^* was cloned in pUASP to generate pUASP- *mtrm^S(124)A^.* The plasmid was used for genetic transformation after confirmation by sequencing.

### Antibodies.

The *mtrm* gene was cloned into a pET-21a vector (Norvagen). 6×His-tagged Mtrm was expressed in the bacterial strain BL21 (DE3), isolated and purified using the Probed Purification System (Invitrogen), and used to raise rabbit and guinea pig polyclonal antisera by Cocalico Biologicals in Reamstown, Pennsylvania, United States. Affinity purification of the antiserum against Mtrm was performed by using a Sulfolink kit from the Pierce Company. Mouse monoclonal antibody to Polo was kindly provided by Moutinho-Santos [13] . Antibody to GFP from rabbits was purchased from Abcam.

### Immunostaining for Mtrm localization.

To prepare ovaries to fix for immunostaining, female fly preparation and ovary dissection were conducted as described in Xiang and Hawley [30]. Whole ovaries were collected and kept in 0.75 ml of 1× Robb's solution during the dissection. After egg chambers were manually teased apart, the ovaries were transferred to an Eppendorf tube. Then, 0.25 ml of 16% formaldehyde was added and incubated for 15 min. The ovaries were washed three times in PBS + 0.1% Triton X-100 (PBST) for 10 min each. After washing three times in PBST, they were incubated in PBST with 5% goat serum for at least 2 h at 4 °C with gentle shaking before being incubated overnight with primary antibodies. Egg chambers were washed four times in PBST and then incubated with proper fluorescently labeled secondary antibodies for 4 h at room temperature. Egg chambers were stained for 10 min in PBST with 0.5 μg/ml DAPI and re-washed four times in the solution for a total of 40 min. The egg chambers were mounted on slides in Vectashield for analysis. Microscopy observation was conducted using a DeltaVision microscopy system (Applied Precision) as described in Xiang and Hawley [30].

### Immunoprecipitations.

To prepare the ovary extract for immunoprecipitation, ovaries from 100 yeast-fed female flies were dissected in 1× PBS. The ovaries were homogenized in an Eppendorf tube at 4 °C by a small pestle in 0.5 ml of ovary extract buffer containing 25 mM Hepes (pH6.8), 50 mM KCl, 1 mM MgCl_2_, 1 mM DTT, and 125 mM sucrose with protease inhibitors cocktail (Calbiochem). The extract was centrifuged at 14,000*g* for 15 min at 4 °C, and the supernatant was collected.

Protein A agarose beads were used for binding polyclonal antibodies from rabbit and guinea pig. Protein G agarose beads were used for binding monoclonal antibody from mouse. 50 μl of protein A- or G-coated agarose was washed three times with PBST (PBS + 0.1% Triton X-100). 10 μl of antibody was added to the beads in a final volume of 500 μl of PBS and mixed on a shaker for 1 h at 4 °C. The beads then were washed twice with PBST. The ovary extract was immunoprecipitated with the beads for 1 h at 4 °C with continual shaking. After recovery by centrifugation at 1,000*g* for 3 min, the beads were washed four times with the cold ovary extract buffer with protease inhibitors, for 5 min each. For Western blotting, the beads were suspended in 30 μl of SDS loading buffer (50 mM Tris-HCl (pH6.8), 100mM DTT, 2% SDS, 0.1% bromophenol blue, 10% glycerol) and heated for 3 min at 95 °C before being loaded on a PAGE gel. Western blotting for Mtrm (Figure 2B) was conducted by using antibody to Mtrm from guinea pigs and an Alkaline Phosphatase chromogen kit (BCIP/NBT) (Roche). Fluorescent Western blotting techniques were used to display both Mtrm and Polo from coimmunoprecipitates on the same membrane.

### Affinity purification of Mtrm-FLAG(3×) from ovaries.

In order to prepare a C-terminally 3× FLAG-tagged Mtrm for the MudPIT mass spectrometry assay, the UASP-*mtrm-Flag* (3×) construct was expressed in ovaries under the control of the *nanos-GAL4* driver in a wild-type background. The extraction of protein from the ovaries was the same as described above. 100 μl of anti-FLAG beads were washed two times with prechilled 1× PBS and then two times with prechilled ovary extract buffer. The anti-FLAG beads were mixed with the extract supernatant, incubated, and washed as described above. After washing, the beads bound with Mtrm-FLAG (3×) were finally transferred to a minicolumn and washed with 25 ml of TBS (50 mM Tris-HCl, pH7.4, 150 mM NaCl) at 4 °C. When washing was completed, 300 μl TBS with 100 μg/ml 3× FLAG peptide was added to elute proteins. TCA was added to the eluted protein solution at a final concentration of 20%. The solution was mixed and kept on ice for at least 30 min. The solution was centrifuged at 14,000 rpm at 4 °C for 15 min. The pellet was collected and 300 μl of prechilled acetone was gently added. After centrifuging again at 14,000 rpm at 4 °C for 15 min, the pellet was carefully collected. The pellet was air dried and ready for the MudPIT spectrometry analysis.

### MudPIT analysis.

TCA-precipitated proteins were urea-denatured, reduced, alkylated, and digested with endoproteinase Lys-C (Roche) followed by modified trypsin (Promega) as described in Washburn [15]. Peptide mixtures were loaded onto 100-μm fused silica microcapillary columns packed with 5-μm C_18_ reverse phase (Aqua, Phenomenex), strong cation exchange particles (Partisphere SCX, Whatman), and reverse phase [42]. Loaded microcapillary columns were placed in line with a Quaternary 1100 series HPLC pump (±Agilent) and a LTQ linear ion trap mass spectrometer equipped with a nano-LC electrospray ionization source (ThermoFinnigan). Fully automated 10-step MudPIT runs were carried out on the electrosprayed peptides, as described in [43]. Tandem mass (MS/MS) spectra were interpreted using SEQUEST [44] against a database consisting of 17,348 D. melanogaster proteins (nonredundant entries downloaded from the National Center for Biotechnology Information [NCBI] 28 November 2006 release), and 177 usual contaminants (such as human keratins, IgGs, and proteolytic enzymes).

To estimate false discovery rates (FDR), each nonredundant protein entry was randomized, keeping the same amino acid composition and length, doubling the search space to a total of 35,050 amino acid sequences (17,525 forward + 17,525 shuffled sequences). Peptide/spectrum matches were selected and compared using DTASelect/CONTRAST [45] with the following criteria set: spectra/peptide matches were only retained if they had a DeltCn of at least 0.08, and a minimum XCorr of 1.8 for singly-, 2.0 for doubly-, and 3.0 for triply-charged spectra. In addition, peptides had to be fully tryptic and at least seven amino acids long. Combining all runs, proteins had to be detected by at least two such peptides or one peptide with two independent spectra. Under these criteria, the average FDR was 0.34 ± 0. To estimate relative protein levels, NSAFs were calculated for each nonredundant protein, as described by Paoletti [16] and Zybailov [17]. Log-transformed NSAF values for proteins reproducibly detected in all three analyses were subjected to a two-tailed *t*-test to highlight proteins significantly enriched in the Mtrm purifications as opposed to negative controls as in Zybailov [17]. A differential modification search was set up to query a protein database containing only the sequences for Mtrm and Polo for peptides containing phosphorylated serines, threonines, tyrosines, and oxidized methionines, i.e., SEQUEST “ASFP” (all spectra against few proteins). The maximum number of modified amino acids per differential modification in a peptide was limited to four.

After this search, an in-house developed script—*sqt-merge* [46]—was used to combine the sets of SEQUEST output files (sqt files) generated from the normal “ASAP” search (all spectra all proteins; i.e., without modifications) and the phosphorylation “ASFP” search described above into one set. This merging step allowed only the best matches to be ranked first. The peptide matches contained in the merged sqt files were compiled and sorted using DTASelect [45]. For the third round of searches, spectra matching modified peptides were selected if they passed the conservative filtering criteria: minimum XCorr of 1.8 for +1, 2.0 for +2, and 3.0 for +3 spectra, with a maximum Sp rank of ten, and fully tryptic peptides with a minimum length of seven amino acids. Xcorr scores for isopeptides, in which any of several adjacent residues could be modified, tend to close, resulting in low normalized differences in Xcorrs. The DeltaCn cut-off was hence set at 0.01 to allow such peptides to be further examined (“–m 0 –t 0 – Smn 7 –y 2 –s 10 −2 2 −3 3 –d 0.01” DTASelect parameters). The coordinates for these spectra were written out into smaller ms2 files using the “– copy” utility of DTASelect. Because these subsetted ms2 files contained, at best, a few hundred MS/MS spectra, they can be subjected to the same phosphorylation differential search against the complete *Drosophila* database (SEQUEST “MSAP,” modified spectra against all proteins). This step allowed us to check that spectra matching modified peptides from Polo and Mtrm sequences did not find a better match against the larger protein database. Again, *sqt-merge* was used to bring together the results generated by these different searches.

DTASelect was used to create reports listing all detected proteins and modified residues on Polo and Mtrm. All spectra matching modified peptides were visually assessed and given an evaluation flag (Y/M/N, for yes/maybe/no). The “no” matches were removed from the final data (-v 2 parameter in DTASelect). Results from different immunoprecipitations were compared using CONTRAST. *NSAF5* (an in-house software by Tim Wen) was used to create the final report on all detected proteins across the different runs, calculate their respective NSAF values, and estimate false discovery rates (FDR). *U_SPC6* software (also in-house by Tim Wen) was used to extract total and modified spectral counts for each amino acid within the proteins of interest and calculate modification levels based on local spectral counts.

### Determining the timing of NEB.

To investigate the timing of NEB, 3-d-old females were collected and fed on yeast for two days. Ovaries were dissected in halocarbon oil 700 (Sigma) on a slide, and egg chambers were separated by mixing using a metal rod. Then, a coverslip was gently put on without pressing and mounting. After waiting for 20–30 min, the egg chambers were observed by phase contrast microscopy in dark view.

### Examining karyosome structure before and after NEB.

To facilitate live imaging of the karyosome before and during NEB, stage 11–12 oocytes from well-fed females were dissected in halocarbon oil and then co-injected with Oli-Green Dye (Molecular Probes) to visualize DNA and Rhodamine-conjugated tubulin (Cytoskeleton) to visualize the spindle and to determine timing of the NEB. Oocytes with germinal vesicles were imaged using a LSM 510 META microscope (Zeiss). Images were acquired using the AIM software v 4 by taking a 10-series Z-stack at 1-μm intervals.

### In situ hybridization.

The 1.686 satellite sequences (also known as the 359-bp repeats) on the *X* chromosome and AATAT repeats on the *4th* chromosome were chosen as probes for in situ hybridization [29,30,47]. The 359-bp sequence of the 1.686 satellite sequences and (AATAT)_6_ repeats were used for probe preparation. Alexa Fluor 488 dye was used for probes of 359-bp sequence on the *X* chromosome. For probes (AATAT)_6_ on the *4th* chromosome, Alexa Fluor 647 dye was used. The details of probe generation and labeling, egg chamber dissection and fixation, fluorescent in situ hybridization, and microscopy observation were described previously [30]. In all oocytes examined for centromere co-orientation, *4* ^th^ chromosomes were observed as red masses of hybridization, whereas the *X* chromosomes were observed as single bright green masses of hybridization. The *FM7* balancer chromosome displays two green blocks of hybridization because of multiple inversions [30]. The AATAT probe is slightly hybridized with an *X* and *FM7* balancer around the centromere region, and therefore both *X* and *FM7* have a slight red signal at centromere location.

## Supporting Information

Figure S1 Expression of Mtrm and Polo in the Later Stages of Oogenesis Formaldehyde-fixed egg chambers in wild type, *w^1118^*, were used for coimmunolocalization of Mtrm and Polo with the polyclonal antibody to Mtrm from a guinea pig and the monoclonal antibody to Polo from mouse. The Mtrm signal is green and the Polo signal is red. As shown in Figure 2, from stages 4–10, Mtrm is mainly localized in the nuclei of both oocytes and nurse cells. However, from stages 10–12, Mtrm is present in high quantities in the oocyte cytoplasm as well. However, the quantity of Mtrm decreases markedly at stage 13. Polo expression begins at stages 11–12 and is maximal by stage 13. However, Polo is localized in cytoplasm of oocytes and is not abundant in the oocyte nucleus. GV indicates germinal vesicle of the oocyte. Scale, 40 μm.
(6.0 MB PDF)

Figure S2 Mtrm Coimmunoprecipitates with Polo Using Antibodies Directed against Polo Mtrm coimmunoprecipitates with GFP-Polo with an antibody to GFP using ovary extracts of GFP-Polo flies (lane 1). Mtrm coimmunoprecipitates with Polo with an antibody to Polo using ovary extracts of wild-type (*w^1118^*) flies (lane 2).
(354 KB PDF)

Video S1 Maintenance of the Karyosome after NEB in Wild-Type Oocytes Live imaging of the karyosome structure after NEB in *FM7/X; mtrm^+^/mtrm^+^* wild type. The movie is taken approximately every 54 s and shown with a display rate of 2 frames/s. Scale, 5 μm.
(7.5 MB AVI)

Video S2 Dissolution of the Karyosome after NEB in *mtrm/+* Oocytes Live imaging of the karyosome structure during NEB in *FM7/X; mtrm^126^/mtrm^+^* oocytes. The movie is taken approximately every 42 s and shown with a display rate of 2 frames/s. Scale, 5 μm.
(9.3 MB AVI)

Video S3 Suppression of the Karyosome Maintenance Defect by Simultaneous Heterozygosity for *polo*
Live imaging of the karyosome structure during NEB in *FM7/X; mtrm^126^ polo^+^/mtrm^+^ polo^16–1^* oocytes. The movie is taken approximately every 54 s and shown with a display rate of 2 frames/s. Scale, 5 μm.
(6.2 MB AVI)

### Accesssion Numbers

 The FlyBase (http://flybase.bio.indiana.edu) accession numbers for genes and gene products discussed in this paper are: *matrimony* (*mtrm*) gene (FBgn0010431); *polo* (FBgn0003124); *twine* (FBgn0002673); and Greatwall/Scant kinase (FBgn0004461).

The Yeast Resource Center (http://www.yeastrc.org/pdr/pages/front.jsp) accession number for the S. pombe Polo homolog *plo1* is CAB11167.

## Abbreviations

FDR: false discovery rate, FISH, fluorescent in situ hybridization
GFP: green fluorescent protein
MudPIT: multidimensional protein identification technology
NEB: nuclear envelope breakdown
NSAF: normalized spectral abundance factors
PBD: Polo-Box Domain
SC: synaptonemal complex
