# Supplementary material for: The Inhibition of Polo Kinase by Matrimony Maintains G2 Arrest in the Meiotic Cell Cycle
Source: PLoS Biol. 2007 Dec 4;5(12):e323. doi: 10.1371/journal.pbio.0050323 (PMC2100146; doi:10.1371/journal.pbio.0050323)
Supplement: Supplementary file 1 [file pbio.0050323.sg001.pdf]

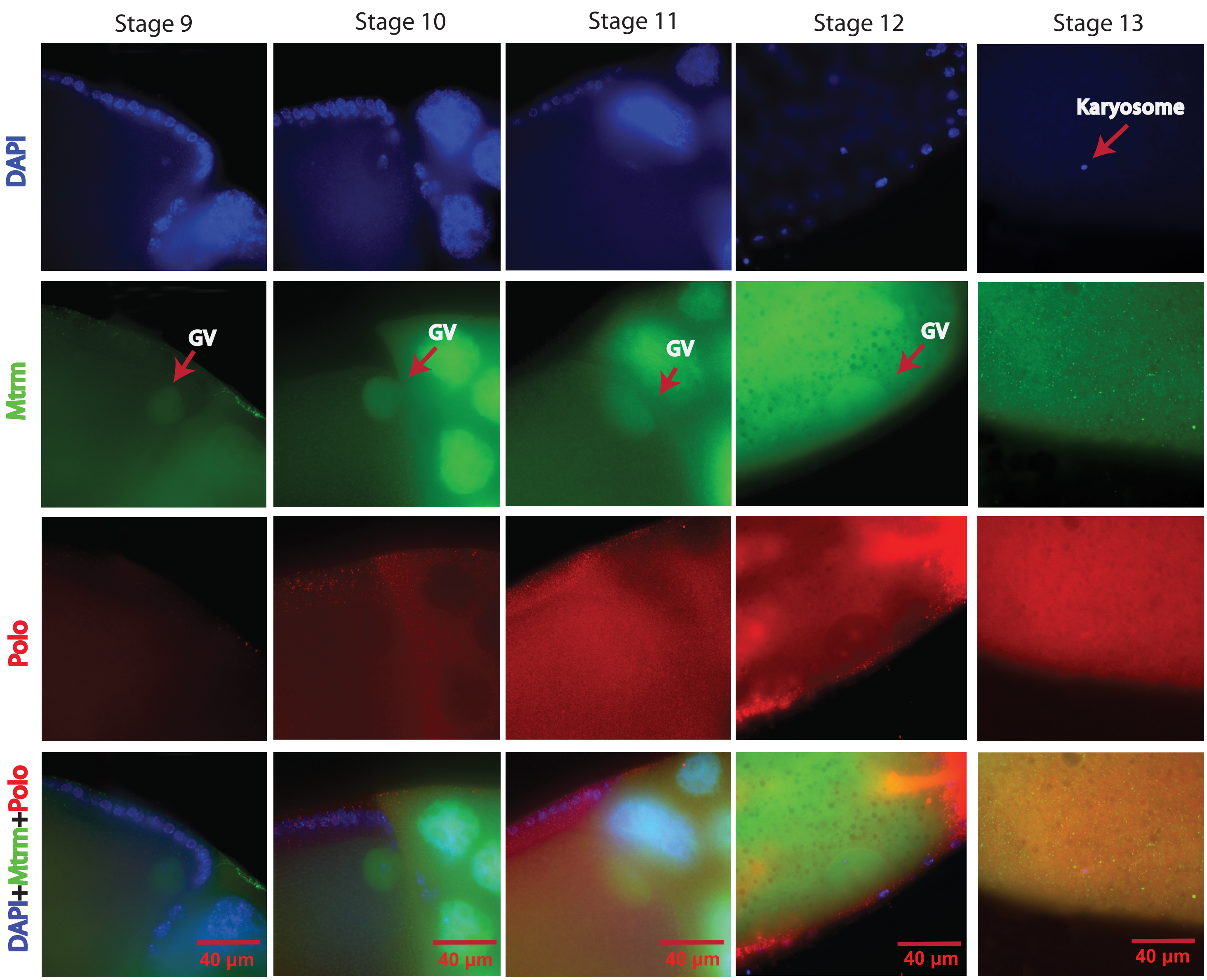

Suppl. Figure 1
